# Fully endoscopic microvascular decompression for the treatment of hemifacial spasm, trigeminal neuralgia, and glossopharyngeal neuralgia: a retrospective study

**DOI:** 10.1186/s12893-023-02214-0

**Published:** 2023-10-27

**Authors:** Weicheng Peng, Rui Zhao, Feng Guan, Xin Liang, Bei Jing, Guangtong Zhu, Beibei Mao, Zhiqiang Hu

**Affiliations:** 1 Department of Neurosurgery, Beijing Shijitan Hospital, Capital Medical University, No.10 Tieyi Road, Haidian District, Beijing, 100038 China; 2 Department of Neurosurgery, Peking University Ninth School of Clinical Medicine, No. 10, tieyi road, Yangfangdian, Haidian district, Beijing, 10038 China

**Keywords:** Endoscopy, Microvascular decompression, Hemifacial spasm, Trigeminal neuralgia, Glossopharyngeal neuralgia

## Abstract

**Background:**

Microvascular decompression (MVD) is already the preferred surgical treatment for medically refractory neurovascular compression syndromes (NVC) such as hemifacial spasm (HFS), trigeminal neuralgia (TN), and glossopharyngeal neuralgia (GPN). Endoscopy has significantly advanced surgery and provides enhanced visualization of MVD. The aim of this study is to analyze the efficacy and safety of fully endoscopic microvascular decompression (E-MVD) for the treatment of HFS, TN, and GPN, as well as to present our initial experience.

**Materials and methods:**

This retrospective case series investigated fully E-MVD performed in 248 patients (123 patients with HFS, 115 patients with TN, and 10 patients with GPN ) from December 2008 to October 2021 at a single institution. The operation duration, clinical outcomes, responsible vessels, intra- and postoperative complications, and recurrences were recorded. Preoperative and immediate postoperative magnetic resonance imaging (MRI) and computerized tomography (CT) were performed for imageological evaluation. The Shorr grading and Barrow Neurological Institute (BNI) pain score were used to evaluate clinical outcomes. The efficacy, safety, and risk factors related to the recurrence of the operation were retrospectively analysed, and the surgical techniques of fully E-MVD were summarised.

**Results:**

A total of 248 patients (103 males) met the inclusion criteria and underwent fully E-MVD were retrospectively studied. The effective rate of 123 patients with HFS was 99.1%, of which 113 cases were completely relieved and 9 cases were significantly relieved. The effective rate of 115 patients with TN was 98.9%, of which 105 cases had completely pain relieved after surgery, 5 cases had significant pain relieved, 4 cases had partial pain relieved but still needed to be controlled by medication. The effective rate of 10 patients with GPN was 100%, 10 cases of GPN were completely relieved after surgery. As for complications, temporary facial numbness occurred in 4 cases, temporary hearing loss in 5 cases, dizziness with frequent nausea and vomiting in 8 cases, headache in 12 cases, and no cerebral hemorrhage, intracranial infection, and other complications occurred. Follow-up ranged from 3 to 42 months, with a mean of 18.6 ± 3.3 months. There were 4 cases of recurrence of HFS and 11 cases of recurrence of TN. The other effective patients had no recurrence or worsening of postoperative symptoms. The cerebellopontine angle (CPA) area ratio (healthy/affected side), the length of disease duration, and the type of responsible vessels are the risk factors related to the recurrence of HFS, TN, and GPN treated by fully E-MVD.

**Conclusions:**

In this retrospective study, our results suggest that the fully E-MVD for the treatment of NVC such as HFS, TN, and GPN, is a safe and effective surgical method. Fully E-MVD for the treatment of NVC has advantages and techniques not available with microscopic MVD, which may reduce the incidence of surgical complications while improving the curative effect and reducing the recurrence rate.

**Supplementary Information:**

The online version contains supplementary material available at 10.1186/s12893-023-02214-0.

## Background

Hemifacial spasm (HFS), trigeminal neuralgia (TN), and glossopharyngeal neuralgia (GPN) are the most common neurovascular compression syndromes (NVC) in clinical practice. The symptoms are closely related to patients’ quality of life and often cause pain and discomfort [1–3].

HFS is one of NVC characterised by intermittent involuntary twitching of muscles innervated by the facial nerve (FN) with an incidence of 1/100,000 [4]. Typical symptoms of HFS begin with involuntary eyelid blinking and gradually progress to buccal muscle twitching, mouth twitching [5]. TN is one of NVC manifesting as neuropathic facial pain, defined by the International Pain Society as “sudden, severe and transient periodic tingling of the skin in the area innervated by one or more branches of the trigeminal nerve (TGN)”. Typical clinical features of TN include paroxysmal pain, remission, precipitating factors and trigger points, etc. The annual incidence of TN is 4.5–28.9/100,000, which is more common in middle-aged women, with the right side more common than the left [6]. GPN is characterised by intermittent, transient, intense and sharp pain in the back of the throat, the base of the tongue, the fossa of the tonsil and the inner ear canal. Sometimes, these episodes of pain can be associated with cardiovascular symptoms, leading to life-threatening episodes of syncope. The annual incidence of GPN is 0.2–0.7/100,000 [7].

The pathogenesis of the above diseases is still unclear, but it is generally believed that the FN, TGN and glossopharyngeal nerves are compressed by peripheral blood vessels (neurovascular compression), leading to nerve demyelination. At present, microvascular decompression (MVD) is already the preferred surgical treatment for NVC such as HFS, TN, and GPN [8]. In recent years, with the increasingly mature application of neuroendoscopic surgical techniques in neurosurgery, more and more studies have been reported on the treatment of HFS, TN, and GPN by endoscopy-assisted MVD, but there are few reports on MVD with the full endoscope [9]. From December 2008 to October 2021, 248 patients with NVC (123 patients with HFS, 115 patients with TN, and 10 patients with GPN) were treated with fully E-MVD in the Department of Neurosurgery, Beijing Shijitan Hospital Affiliated to Capital Medical University, and the clinical therapeutic effect was satisfactory. This study reports on the efficacy and safety of fully E-MVD for the treatment of HFS, TN, and GPN, as well as our surgical experience and skills.

## Materials and methods

### Patient recruitment

This single-centre, retrospective study was conducted in accordance with the Helsinki Declaration of 1975, as revised in 2000. This study was approved by the medical ethics committee of Beijing Shijitan Hospital, Capital Medical University, China. All patients or their legal guardians in this study authorised the release of their medical records and information.

This study included the clinical data of 248 patients with NVC (such as HFS, TN, and GPN) who underwent E-MVD at the Department of Neurosurgery, Beijing Shijitan Hospital, affiliated with Capital Medical University, from December 2008 to October 2021. All eligible patients enrolled in this study met the following criteria: (1) The patient was consistent with the clinical diagnosis of HFS, TN, and GPN; (2) Patients were excluded from secondary HFS, TN, and GPN (such as tumours, arteriovenous malformations compressing nerves, etc.); (3) Patients with multiple sclerosis HFS, TN, and GPN were excluded; (4) The patient had no serious systemic disease and could tolerate anaesthesia and surgery.

The Shorr grading was used to assess clinical outcomes in patients with HFS, while the BNI pain score was used in patients with TN or GPN. According to Shorr grading, grade 0: no spasm; grade I: increased blinking on external stimulation; grade II: mild, facial muscle slight tremor, no dysfunction; grade III: moderate, facial muscle spasm obvious, mild dysfunction; grade IV: severe, severe spasm with small eye cracks and severe dysfunction (inability to walk, read, etc.) [10]. The criteria used to assess postoperative efficacy for TN and GPN patients is the Barrow Neurological Institute (BNI) pain score, where grade I is complete pain relief, grade II is most pain relief and no need for low-dose medication control, grade III is partial pain relief that can be controlled with medication, grade IV is partial pain relief that cannot be controlled with medication, and grade V is no pain relief [11].

### Imaging data

**Fig. 1:**
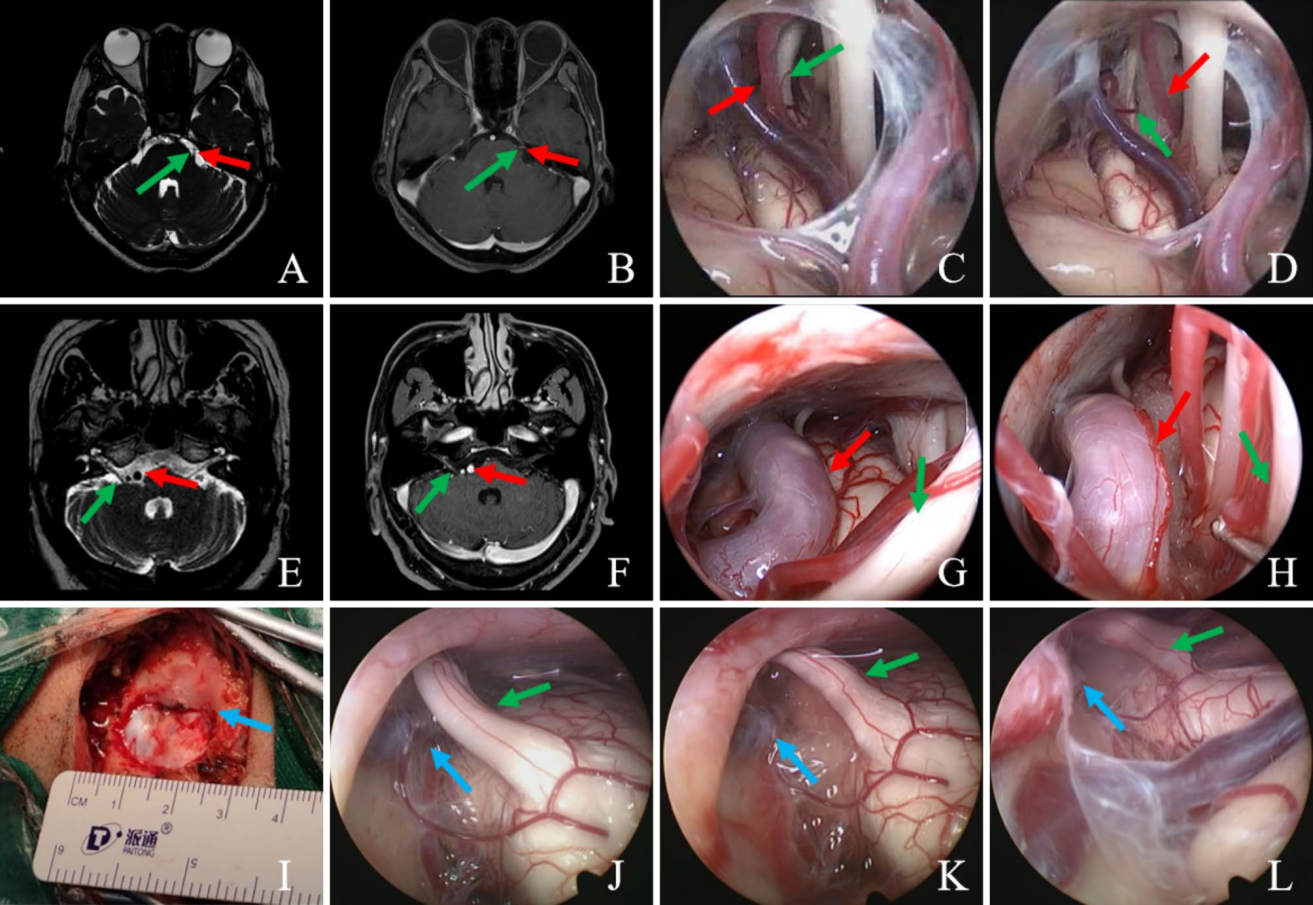
**A-D, A case of TN.** **(A-B)**: Images A and B show the patient’s preoperative 3D FIESTA MRI and 3D-TOF MRA imaging, respectively, with the green arrow pointing to the trigeminal REZ and the red arrow pointing to the responsible vessel, which forms a compression in the REZ of the TGN; **(C-D)**: Pictures C and D show that the responsible artery (indicated by the red arrow) forms a neurovascular compression from the trigeminal nerve (indicated by the green arrow) REZ to Meckel’s bursa throughout, and that the neurovascular decompression is adequate and definitive with the aid of the endoscope with a Teflon cotton placed under direct vision. **E-H, A case of HFS.** **(E-F)**: Images E and F show the patient’s preoperative 3D FIESTA MRI and 3D-TOF MRA imaging, respectively. The dilated displaced basilar artery (indicated by the red arrow) squeezes the ipsilateral nerve (indicated by the green arrow); **(G-H)**: Thick basilar artery (indicated by the red arrow) squeezing the REZ of the FN (indicated by the red arrow), with high tension between them and the brainstem. Intraoperatively, the basilar artery is cushioned away from the FN using “pre-placed” technique and “set up bridge” technique to achieve adequate decompression. **I, Surgical incision and bone window.** The surgical design creates an elliptical microbone window of approximately 2.5 cm in diameter, revealing the junction of the transverse and sigmoid sinuses (indicated by the blue arrow). **J-L, A case of TN with venous-type compression.** Intraoperatively, the responsible vein (indicated by the blue arrow) was seen to form a compression beneath the TGN (indicated by the green arrow), which was adequately decompressed by the operator using “pre-placed” technique, and the decompression was checked by “diving” technique at the end of the procedure

CT and MRI were performed in all cases prior to E-MVD to exclude patients with HFS, TN and GPN caused by multiple sclerosis and neoplastic lesions. According to the results, the surgeon further clarified the relationship between the patient’s responsible vessels and the corresponding cranial nerves, developed a more reasonable surgical programme and improved the identification rate of the responsible vessels. MRI identified the diagnose of HFS, TN and GPN [Fig. 1. A, B, E and F]. Immediate postoperative CT and MRI were performed to rule out complications such as cerebral haemorrhage and cerebral contusion.

### Surgical procedure

After satisfactory general anaesthesia, all patients were placed in the lateral decubitus position. The post-sigmoid keyhole approach was adopted, and a straight incision of approximately 4 cm was made at the post-mastoid hairline, starting 1 cm above the transverse sinus, and a microbone window craniotomy was performed to create an elliptical bone window with a diameter of 2.0 ~ 3.0 cm [Fig. 1. I]. TGN decompression was required to expose the junction of the transverse sinus and sigmoid sinus, while facial, glossopharyngeal and vagus nerve decompression was required to expose inferiorly. The dura was cut in a “K” shape and the free edge was suspended and fixed. Sufficient cerebrospinal fluid (CSF) was released during surgery to allow natural collapse of the cerebellar hemispheres. The 30° endoscope was slowly inserted along the side of the cerebellum after covering the cerebellum with brain cotton.

The operator introduced the endoscopic lens into the CPA region along the petrous bone or the junction between the petrous bone and the canopy to first understand the relationship of each nerves and vessels in the visual field and to judge the surgical path. The root entry/exit zone (REZ) of the FN, TGN, glossopharyngeal nerve and vagus nerve were clearly observed and the responsible vessels were explored. The REZ of the FN should be comprehensively observed during FN decompression, while the whole process from the REZ to the Meckel’s sac should be observed during TGN decompression [12]. The endoscopic view allows the operator to dissect the arachnoid, expose the nerve and vessels, and after dissecting the responsible vessels, completely release the arachnoid around the nerve and blood vessels.

The operator can use the “pre-placed” technique, i.e. using the “lever principle”, pre-place 1–2 small pieces of Teflon cotton at the proximal end of the responsible vessel (the responsible vessel is above) or the nerve (the responsible vessel is below) to lift it appropriately to relieve the pressure at the neurovascular compression point, then place the Teflon cotton at the compression point to release the compression and withdraw the pre-pad after the position is satisfactorily adjusted. In cases where the vertebrobasilar artery is collaterally compressed or is pushing other vessels to compress the nerve, the operator can use the “set up bridge” technique, in which two small cotton pads are placed on either side of the vertebrobasilar collaterals to properly elevate them away from the brainstem, and then Teflon cotton is sequentially placed at the point of contact between the responsible vessel and the nerve to relieve the compression. In the case of a developed petrous bone, the endoscopic lens is inserted from above and the surgical instrument from below, without grinding the petrous bone. Finally, the operator uses the “diving” technique to mimic brain pulsation in the physiological state to check the decompression effect and ensure that the Teflon cotton pad does not move, while keeping the surgical field clean and replacing bloody CSF and air to avoid postoperative adhesions. Before the end of the operation, the operator should observe the operative field in several directions and comprehensively to avoid missing the responsible vessels. Parts of the surgical process are shown in Fig. 1.

As for responsible veins, We should never consider coagulation or sacrifice of the involved veins, even in sophisticate situations of neurovascular compression. For large veins, like the trunk of the PV, which are frequently seen in parallel, riding, or even twisting compression, the interpose method can occasionally be added to the transpose method, which uses a Teflon cotton sling attached to the petrosal dura and requires the use of medical adhesive. For medium size veins, like the pontotrigeminal veins, which usually manifest as moderate compression, direct interposition of Teflon cotton may be sufficient. Small perforator veins were also not sacrificed, but a small Teflon cotton was inserted. Last but not least, in the extreme situation where sufficient decompression of the responsible veins was not possible, nerve combing of the sensory root of the TGN was performed.

### Discharge and follow-up assessment

This study used outpatient visits, inpatient observation and telephone follow-up for follow-up. Follow-up included short-term and long-term postoperative symptom improvement, medication use, relapse, and other neurological complications.

In this study, the surgical efficacy of HFS patients was evaluated according to preoperative and postoperative Shorr grading. A postoperative Shorr grading of 0 was considered cured, a postoperative Shorr grading decrease of ≥ 1 compared to preoperative was considered effective, and no significant postoperative change was considered ineffective. The criteria used to assess postoperative efficacy for TN and GPN patients is the Barrow Neurological Institute (BNI) pain score. In this study, BNI grade I was considered cured, BNI grade II was considered excellently effective, BNI grade III was considered effective, and BNI grades IV-V were considered ineffective.

### Data analysis

All data were analyzed using statistical software (SPSS version 19.0; IBM Corp, Armonk, NY, USA). Continuous variables are presented as mean ± standard deviation. Counts or ranked variables are presented as mean (range, minimum-maximum). Statistical analysis was performed using generalised linear models. The Chi-square test or Fisher’s exact test were used for dichotomous variables.

## Results

### Demographic and baseline characteristics

A total of 248 patients (103 males) met the inclusion criteria were retrospectively studied. This study included the clinical data of 248 patients with NVC (such as HFS, TN, and GPN) who underwent E-MVD at the Department of Neurosurgery, Beijing Shijitan Hospital, affiliated with Capital Medical University, from December 2008 to October 2021.

There were 123 patients with HFS, including 49 males and 74 females; age ranged from 24 to 78 years, with a mean of 49.5 ± 11.1 years. The disease duration ranged from 0.5 to 7.3 years, with a mean of 3.2 ± 0.1 years. All symptoms were unilateral, including 81 cases on the right side (65.9%) and 42 cases on the left side (34.1%). According to Shorr grading, 12 cases were grade II, 108 cases were grade III, and 3 cases were grade IV.

There were 115 patients with TN, 46 males and 69 females; age ranged from 20 to 82 years, with a mean of 60.0 ± 11.8 years. The disease duration ranged from 1.1 to 8.0 years, with a mean of 3.5 ± 0.5 years. The pain was located on the left side in 52 cases (45.2%) and on the right side in 63 cases (54.8%). The pain involved the V1 branch of the trigeminal nerve in 12 cases (10.4%), the V2 branch in 23 cases (20.0%), the V3 branch in 18 cases (15.7%), both V1 and V2 branches in 19 cases (16.5%), both V2 and V3 branches in 34 cases (29.6%) and both V1, V2 and V3 branches in 9 cases (7.8%).

There were 10 patients with GPN, including 8 males and 2 females, age ranged from 37 to 64 years, with a mean of 54.5 ± 13.0 years. The disease duration ranged from 1.1 to 4.0 years, with a mean of 2.6 ± 0.3 years. All patients had varying degrees of pharyngeal pain, including 2 cases radiating deep into the external auditory canal and 4 cases radiating to the base of the tongue. The demographics and baseline characteristics were showed in Table 1.

**Table 1:** Demographic and baseline characteristics (n = 248)

Variable	Median (IQR) in years/no of patients (%)
HFS	
Total no. of patients	123
Age	49.5土11.1
Women	74 (60.1%)
Laterality Lt	42 (34.1%)
Shorr grading	
Shorr 0	0 (0)
Shorr I	0 (0)
Shorr II	12 (9.8%)
Shorr III	108 (87.8%)
Shorr IV	3 (2.4%)
Mean duration of Sx prior to op, yrs	3.2 ± 0.1
TN	
Total no. of patients	115
Age	60.0土11.8
Women	69 (60.0%)
Laterality Lt	52 (45.2%)
Trigeminal nerve pain distribution (%)	
V1 only	12 (10.4%)
V2 only	23 (20.0%)
V3 only	18 (15.7%)
V1&V2	19 (16.5%)
V2&V3	34 (29.6%)
V1,V2&V3	9 (7.8%)
Mean duration of Sx prior to op, yrs	3.5土0.5
GPN	
Total no. of patients	10
Age	54.5土13.0
Women	2 (20.0%)
Pain location	
Pharyngeal	8 (80.0%)
External ear canal	2 (20.0%)
Base of the tongue	4 (40.0%)
Mean duration of Sx prior to op, yrs	2.6土0.3

HFS: hemifacial spasm; TN: trigeminal neuralgia; GPN: glossopharyngeal neuralgia

### Evaluation of clinical outcomes

All surgeries were successfully performed. The effective rate of 123 patients with HFS was 99.1%, of which 113 cases were completely relieved (including 5 cases of delayed healing) and 9 cases were significantly relieved. 113 patients recovered from preoperative grade II and III to grade 0, 7 patients recovered from grade III to grade I, 2 patients recovered from grade IV to grade II, and 1 patient had no significant relief from grade IV. The effective rate of 115 patients with TN was 98.9%, of which 105 cases had complete postoperative pain relief and reached BNI grade I.5 cases had significant pain relieved, and did not need medication for pain control and reached BNI grade II. 4 cases had partial pain relieved but still needed to be controlled by medication, but the dose was significantly reduced compared with the preoperative dose, reaching BNI grade III. 1 case had no pain relief and was classified as BNI grade IV-V. The effective rate of 10 patients with GPN was 100%, 10 cases of GPN were completely relieved after surgery. The mean operative duration was 111.2土54.3 min and the mean hospital stay was 14.4土4.7 days. The evaluation of clinical outcomes were showed in Table 2.

**Table 2:** The evaluation of clinical outcomes (n = 248)

	Value	
	Pre-op	Post-op
HFS		
Shorr 0	0	116
Shorr I	0	0
Shorr II	12	2
Shorr III	108	4
Shorr IV	3	1
TN		
BNI I		105
BNI II		5
BNI III		4
BNI IV		1
BNI V		0
GPN		
BNI I		10
BNI II		0
BNI III		0
BNI IV		0
BNI V		0

HFS: hemifacial spasm; TN: trigeminal neuralgia; GPN: glossopharyngeal neuralgia

### Responsible vessel

In 248 patients, responsible vessels were found in 246 patients and local arachnoid thickening was considered in 2 patients.

Responsible vessels were found in 123 patients with HFS. There were 103 cases of simple arterial compression, including 11 cases of superior cerebellar artery, 58 cases of anterior inferior cerebellar artery, 24 cases of posterior inferior cerebellar artery, 4 cases of vertebral artery, and 6 cases of more than two arteries involved.There were 11 cases of arteriovenous compression, of which the superior cerebellar arteries and veins were involved in 6 cases and the anterior inferior and posterior inferior cerebellar arteries and veins were involved in 5 cases. There were 9 cases of simple venous compression, all of which were petrosal vein (PV) and their branches.

In 115 patients with TN, responsible vessels were found in 113 patients and local arachnoid thickening was considered in 2 patients. There were 88 cases of simple arterial compression, including 59 cases of superior cerebellar artery, 10 cases of anterior inferior cerebellar artery, 8 cases of posterior inferior cerebellar artery, 2 cases of basilar artery, 3 cases of vertebral artery, and 6 cases of more than two arteries involved. There were 19 cases of arteriovenous compression, including 12 cases of superior cerebellar arteries and veins, and 7 cases of anterior inferior and posterior inferior cerebellar arteries and veins. There were 6 cases of simple venous compression, all of which were PV and their branches. Arachnoid thickening was found in 2 case without obvious vascular compression.

Responsible vessels were found in 10 patients with GPN, including anterior inferior cerebellar artery in 1 case, posterior inferior cerebellar artery in 6 cases, vertebral artery combined with posterior inferior cerebellar artery in 2 cases, and vein in 1 case.

### Petrosal vein (PV) classification

Through the fully E-MVD procedure in 248 patients, we found that the anatomy of the PV in the endoscopic view has its unique characteristics, and we summarized the preliminary clinical practical staging of the PV in the endoscopic view according to the length and tension.

**Fig. 2:**
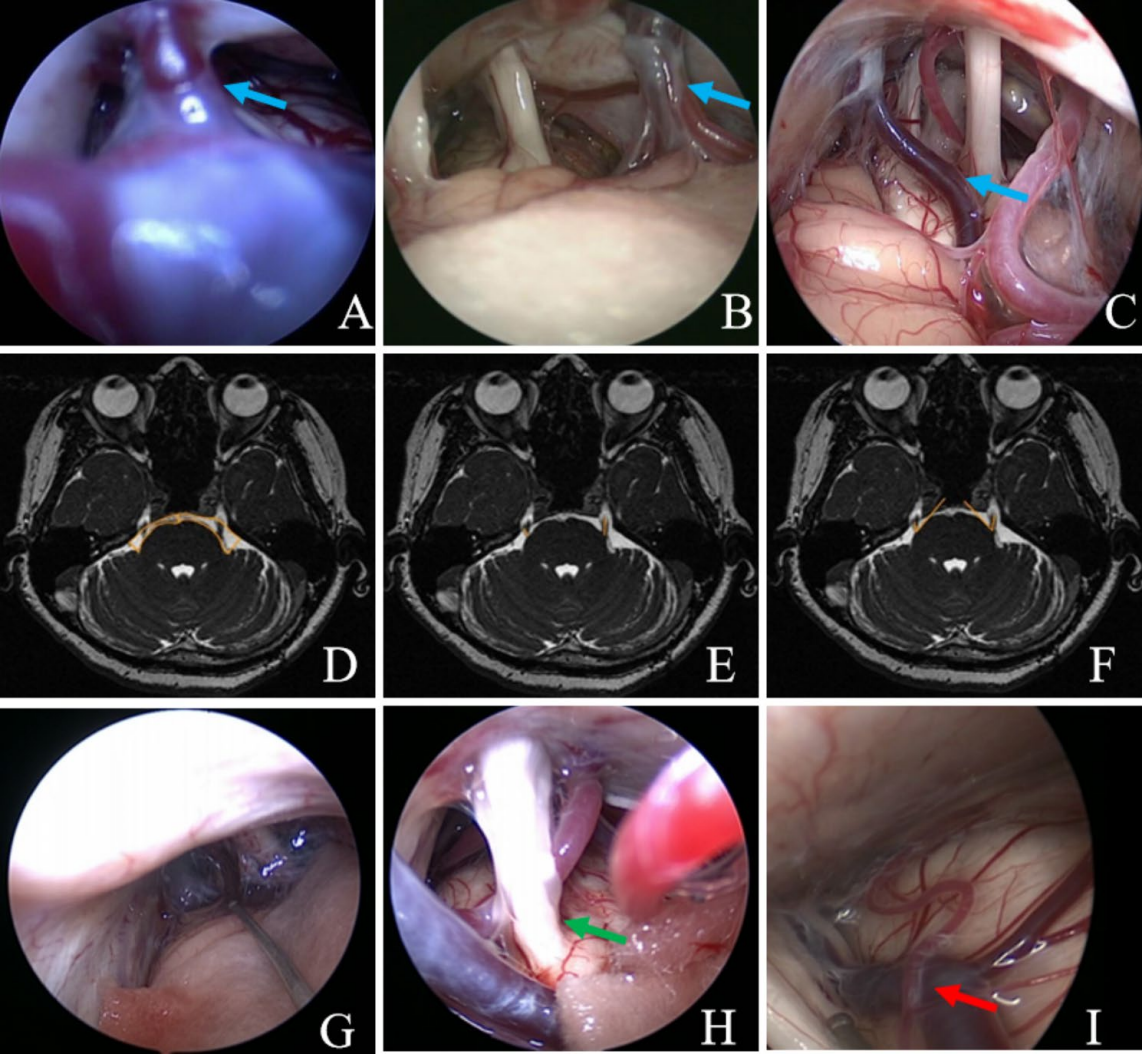
**A-C, Clinical classification of PV (indicated by the blue arrow).** **(A)** Type I: length < 5 mm and the tension is high; **(B)** Type II: length between 5 ~ 10 mm and the tension is moderate; **(C)** Type III : length > 10 mm and the tension is low. **D-F, Preoperative 3D FIESTA MRI of patients with recurrent TN.(D)** Area values of the CPA area bilaterally, the area of the CPA on the left side is larger than that on the right side, the area ratio of the CPA (healthy/afflicted side) = 1.67/0.91 = 1.84 > 1; **(E)** Length values of the TGN bilaterally, it can be seen that the length of the TGN on the left side is longer than that on the right side, the length ratio of the TGN (healthy/afflicted side) = 0.82/0.45 = 1.82 < 1; **(F)** TGN angle values, it can be seen that the angle of the left side is larger than that of the right side, the angle ratio of TGN (healthy/afflicted side) = 51.8/42.4 = 1.22 < 1. **G-I, Intraoperative endoscopic images of the patient with recurrence. (G)** The overall area of the CPA is narrower in the endoscopic field of view, which makes endoscopic manipulation difficult; **(H)** The responsible vessels are visible, forming obvious compression on the TGN, and the green arrow indicates severe deformation of the TGN due to long-term vascular compression; **(I)** The red arrow indicates simultaneous arterial and venous compression of the TGN

PV in the endoscopic view could be roughly divided into types I ~ III and there were 3 types in total. Type I: length < 5 mm and the tension is high [Fig. 2. A]; Type II: length between 5 ~ 10 mm and the tension is moderate [Fig. 2. B]; Type III : length > 10 mm and the tension is low [Fig. 2. C]. Of the 248 patients in our group, there were 21 patients whose PV was classified as type I, 67 patients as type II and 160 patients as type III.

### Complications

Patients experienced temporary facial numbness in 4 cases and temporary hearing loss in 5 cases after surgery, which resolved after nerve nutrition and other treatments. Patients developed dizziness with frequent nausea and vomiting in 8 cases and headache in 12 cases after surgery, which improved after symptomatic treatment during hospitalisation. No patients had complications such as death, stroke, cardiac event, hearing loss, facial paralysis, hemiparesis, CSF leakage, etc. The incidence of surgical and postsurgical complications were showed in Table 3.

**Table 3:** Incidence of surgical and postsurgical complications (n = 248)

Complication	No.(%)
Permanent complications	
Death	0 (0)
Stroke	0 (0)
Cardiac event	0 (0)
Partial hearing loss	0 (0)
Facial paralysis	0 (0)
Paralysis of arms and/or legs	0 (0)
Subjective/transient complications	
Facial numbness	4 (1.6%)
Hearing loss	5 (2.0%)
Transient unsteadiness/dizziness	8 (3.2%)
Headaches	12 (4.8%)
Vision problems	0 (0)
Diplopia	0 (0)
Hydrocephalus requiring shunt	0 (0)
CSF leak	0 (0)

CSF: cerebrospinal fluid

### Follow-up information and risk factors for recurrence

Of the 248 patients, the follow-up period ranged from 3 to 42 months, with a mean of 18.6 ± 3.3 months. There were 15 cases of recurrence among the 246 patients who responded to surgery. Of 122 patients with HFS who responded to surgery, 116 patients had complete remission to grade 0, 2 patients were in grade II (grade IV before surgery), and 4 patients had recurrence (grades II and III before surgery, grade 0 after surgery, and grade III during follow-up), with an overall cure rate of 94.3%. For 114 patients with TN who responded to surgery, 98 patients had complete pain relief, 5 patients had most of the pain relief, and 11 patients had recurrence during follow-up, with an overall cure rate of 86.0%. Of 10 patients with GPN who responded to surgery, all patients had complete pain relief, and none had a recurrence or worsening during follow-up, with an overall cure rate of 100%. These variables were statistically significant in patients with different endpoints, including disease duration, type of responsible vessel, and CPA area ratios (healthy/affected side) showed statistically significant differences between different groups (P < 0.05, Table 4).

**Table 4:** Comparison of postoperative recurrence in NVC patients treated with fully E-MVD

Variables	NVC patients responded to E-MVD (n = 246)		χ² Value	P Value
	Recurrence (n = 15)	Recurrence-free (n = 231)		
Sex, n (%)			0.122	0.582^*^
Female	8 (53.33)	136 (58.87)		
Male	7 (46.67)	95 (41.13)		
Age (years), n (%)			0.032	0.806^*^
≤ 60	9 (60.00)	130 (56.28)		
> 60	6 (40.00)	101 (43.72)		
Disease duration (years), n (%)			3.046	0.047^*^
≤ 3	2 (13.33)	80 (34.63)		
> 3	13 (86.67)	151 (65.37)		
Type of responsible vessel, n (%)			18.091	<0.001^*^
Arterial	2 (13.33)	200 (86.58)		
Non-arterial	13 (86.67)	31 (13.42)		
CPA area ratio, n (%)				0.014^#^
≤ 1	5 (33.33)	161 (69.70)		
> 1	10 (66.67)	70 (43.30)		
Angle ratio of FN/TGN, n (%)			1.651	0.282^*^
≤ 1	4 (26.67)	103 (44.59)		
> 1	11 (73.33)	128 (55.41)		
Length ratio of FN/TGN (cisternal segment), n (%)			0.275	0.639^*^
≤ 1	6 (40.00)	80 (34.63)		
> 1	9 (60.00)	151 (65.37)		

NVC: neurovascular compression syndromes; E-MVD: endoscopic microvascular decompression; CPA: cerebellopontine angle; FN: facial nerve; TGN: trigeminal nerve; ^*^ chi-square test; ^#^ fisher’s exact test

## Discussion

### History of MVD for the treatment of HFS, TN, and GPN

In 1932, Dandy first described the relationship between the superior cerebellar artery and the TGN root and speculated that pain might be related, but he did not make any surgical attempts to separate the vessels from the nerves [13]. In 1959, Gardner performed the first true MVD on a patient with TN and then extended MVD to HFS [14]. Graf-radford et al. pointed out that by establishing the theory of MVD, MVD has become one of the main treatment methods for TN [15]. Since 1967, Jannetta has performed a large number of MVDs and achieved good clinical effects [16]. At the same time, he proposed the concept of MVD and popularised it worldwide. In 1977, Jannetta improved the surgical method based on the original theory, performed microsurgical techniques, and performed MVD with good results [17]. However, a major drawback of MVD surgery is that about 15% of patients cannot accurately find the responsible vessels compressing the TGN during surgery. At this point, amputation of the TGN root is required. This not only exposes the patient to the various risks of craniotomy, but also suffer complete anesthesia after surgery [18].

### Theoretical basis of fully E-MVD

In recent years, MVD has gradually developed and matured in terms of theoretical basis and surgical techniques, but the detection rate of responsible vessels in microscopic MVD is about 89.0% − 95.0%, the surgical efficiency is 81.0% − 86.0%, and the recurrence rate is 5.0% − 14.0%. The reasons for poor surgical efficacy and high postoperative recurrence rate are mainly intraoperative failure to identify responsible vessels, omission of responsible vessels or inadequate decompression [19–21]. Most of these missing responsible vessels are located in the REZ or Makler capsule area, or are blocked by the petrosal bone and become the anatomical blind area in microscopic surgery. It is often necessary to grind away the petrosal tubercle and overstretching the cerebellar hemispheres to barely expose the responsible vessels, which increases the incidence of complications [22].

Gradually, some neurosurgeons have found that endoscopic-assisted microscopic MVD surgery has more advantages in identifying the responsible vessels, so they are performing endoscopic-assisted microscopic MVD surgery for TN. In 2002, Jarrahy first reported the treatment of TN by fully E-MVD [23]. Subsequently, Kabil, in his retrospective study of 255 patients, reported an intraoperative detection rate of 100.0% of responsible vessels for fully E-MVD for TN, with postoperative and 3-year facial pain relief rates as high as 95.0% and 93.0%, respectively, with more similar reports in recent years [19, 20, 24, 25]. In the 248 patients treated with fully E-MVD in this study, the responsible vessel detection rate was 99.2% (246/248), the surgical efficiency was 99.2% (246/248), and the recurrence rate was 6.1% (15/246).

### The advantage of fully E-MVD for treatment of HFS,TN, and GPN

(1) The endoscope provides excellent visualization and comprehensive evaluation of the neurovascular compressions in HFS, TN, and GPN patients [26]. The fully E-MVD procedure allows the operator to have a panoramic view of the CPA area, and by adjusting the depth and angle of the lens, the operative field is revealed completely and clearly, avoiding the blind spots of traditional microscopy. The majority of patients in this group had a single responsible vessel compression, with approximately 17.7% (44/248) having more than two responsible vessels, which further suggests that avoiding missing responsible vessels is necessary to reduce the recurrence rate. In 22.6% (56/248) of our group, the responsible vessel compression was found to be ventral to the corresponding cranial nerve, a location that is often difficult to detect in microscopic MVD. In contrast, endoscopy provides a better view to detect vascular compressions hidden ventral to the corresponding cranial nerve without pulling the brain tissue and nerves. (2) During the fully E-MVD procedure, after the neurovascular compression has been clarified, the operator can pad the Teflon cotton into the ideal position under direct vision, and check whether the ideal position of the Teflon cotton can be achieved, so as to achieve sufficient decompression, consolidate the surgical effect and reduce the recurrence rate. (3) E-MVD utilises the space between the vessels, nerves and surrounding tissues in the CPA without grinding away the petrosal tubercle and overstretching the cerebellar hemispheres, results in less injury and bleeding, reducing the incidence of complications. (4) The “pre-placed” technique used during surgery can effectively prevent accidental injury during sharp neurovascular separation, and it is convenient to adjust the position of the Teflon cotton pad when the operator holds the endoscope in one hand and operates with the other. (5) The “set up bridge” technique can effectively expose the blocked portion of the vertebrobasilar artery and fully decompress the nerve, ensuring that the vertebral or basilar artery does not rebound after decompression, ensuring adequate nerve decompression and reducing the recurrence rate. (6) The “diving” technique, which mimics brain pulsations in a physiological state, checks the decompression effect, ensures that the padded cotton does not move and reduces post-operative recurrence. It also allows intracranial haemorrhage and air to be exchanged, keeping the area clean, reducing complications such as headaches and avoiding post-operative adhesions that lead to recurrence.

### Clinical classification of PV

The PV is anatomically closely related to cranial nerves such as the facial, trigeminal, glossopharyngeal and vagus nerves, and is also an important drainage vein of the posterior fossa [27]. In MVD surgery, the PV often becomes an obstructing vein that prevents exposure of the surgical field and interferes with the surgical procedure [28]. Therefore, the anatomical location of the PV in the endoscopic view directly interferes with the surgical procedure, thus affecting the surgical effect and even leading to complications due to accidental injury. The PV drains blood to most areas of the brainstem and cerebellum, and accidental dissection of this vein during surgery may result in stroke with serious complications such as cerebellar or brainstem oedema and infarction. In 248 patients who underwent fully E-MVD, we found that the anatomy of the PV in the endoscopic view has its own unique characteristics, and we summarized the preliminary clinical practical staging of the PV in the endoscopic view based on its length and tension. There were a total of 21 patients with type I PV in this group, including 3 patients with PV injury and bleeding. Combined with literature reports and clinical experience, PV burning should be avoided during surgery as much as possible to reduce the incidence of complications. According to the practical clinical staging of PV, if the PV is type I, the operator needs to be more careful during the operation to avoid significant lateral adjustment of the scope to prevent damage to the PV and complications of haemorrhage and bruising stroke.The practicality and scientific validity of the clinical classification of PV we have summarized may need to be supported by more case numbers and further studies.

### Recurrence-related factors

With regard to the efficacy of MVD and the risk of long-term recurrence, the literature reports that age, gender and the degree of compression of the responsible vessel are factors that cannot be ignored when assessing the risk of long-term recurrence after surgery [29]. Some scholars believe that patients over 60 years of age have better long-term efficacy, which may be related to brain atrophy and the relatively large posterior fossa space in older patients [30]. It has been reported that female patients have a higher postoperative recurrence rate due to the small volume of the posterior fossa and the higher probability of vaso-nerve contact in CPA [31, 32]. The size of the CPA is a diagnostic aid for HFS, TN and GPN, and is an important factor in the outcome of MVD after surgery, as well as an influential factor in surgical intervention [33, 34]. In our present analysis of clinical data, we found that patients with a smaller ratio of CPA area on the affected side to CPA area on the healthy side were more likely to have recurrence after surgery. We speculated that the main reason for this was that the reduced CPA area resulted in more opportunities for neurovascular contact in this area, increasing the difficulty of performing fully E-MVD [Fig. 2. D-I]. Patients with a short disease duration may have relatively good results after fully E-MVD. Long-term nerve compression is prone to irreversible nerve damage. Even if the neurovascular compression is relieved by surgery, the clinical symptoms are often not easy to recover. The recurrence rate was lower when the artery was the responsible vessel. The recurrence rate is higher when the venous or arteriovenous compression is the responsible vessel. Simple arterial compression is often easier to identify intraoperatively, allowing for more adequate decompression. Venous compression is easily ignored and omitted, affecting the effect of intraoperative decompression and leading to postoperative recurrence. Similar to our previous studies, it is believed that the CPA area ratio (healthy/affected side), the length of disease duration, and the type of responsible vessels are the risk factors related to the recurrence of HFS, TN, and GPN treated by fully E-MVD. [35] Therefore, it is of great importance to fully understand the medical history and review the imaging before surgery to assess the patient’s condition and make surgical plans.

### Study Limitations

The design of the present study was retrospective and nonblinded, which has some limitations. The retrospective study in a single center with limited sample size restricts the strength of the data or conclusion. Selection bias largely deviates this study’s generalizability. The patient sample represented the practice of a single neurosurgeon at a tertiary referral center and therefore may lack generalizability to other practice settings. Follow-up intervals were not standardized. A better study design would have required telephone calls at serial time points after surgery, for example at yearly intervals. Further studies are needed to investigate the efficacy and safety of fully EVD for the treatment of HFS, TN and GPN.

## Conclusions

In this retrospective study, our results suggest that the fully E-MVD for the treatment of NVC such as HFS, TN, and GPN, is a safe and effective surgical method. Fully E-MVD for the treatment of NVC has advantages and techniques not available with microscopic MVD, which may reduce the incidence of surgical complications while improving the curative effect and reducing the recurrence rate.

### Electronic supplementary material

Below is the link to the electronic supplementary material.

Supplementary Material 1

Supplementary Material 2

Supplementary Material 3

## Data Availability

The data analyzed during the current study are available from the corresponding author on reasonable request.

## List of abbreviations

MVD: Microvascular decompression
E-MVD: Endoscopic microvascular decompression
NVC: Neurovascular compression syndromes
HFS: Hemifacial spasm
FN: Facial nerve
TN: Trigeminal neuralgia
TGN: Trigeminal nerve
GPN: Glossopharyngeal neuralgia
MRI: Magnetic resonance imaging
CT: Computerized tomography
CPA: Cerebellopontine angle
REZ: Root entry/exit zone
BNI: Barrow Neurological Institute
PV: Petrosal vein
CSF: Cerebrospinal fluid
